# Supplementary material for: Construction of a Ternary Composite Colloidal Structure of Zein/Soy Protein Isolate/Sodium Carboxymethyl Cellulose to Deliver Curcumin and Improve Its Bioavailability
Source: Foods. 2023 Jul 13;12(14):2692. doi: 10.3390/foods12142692 (PMC10379602; doi:10.3390/foods12142692)

Supplement Figure captions

Supplementary Figure S1 zein NPs under same condition from (5 mg/mL to 20 mg/mL)

Supplementary Figure S2 zein NPs and a series of zein:SPI (Z/S NPs) from (4:1 to 2:5)

Supplementary Figure S3 A series of SPI:CMC-Na (Z/S/C NPs) (4:1 to 2:5)

Supplementary Figure S4 Cur-loaded zein nanoparticle, including Z-cur, Z/S-cur and Z/S/C-cur

Supplementary Figure S5 Enlarged XRD for Z-cur, Z/S-cur, Z/S/C-cur

Supplementary Figure S1

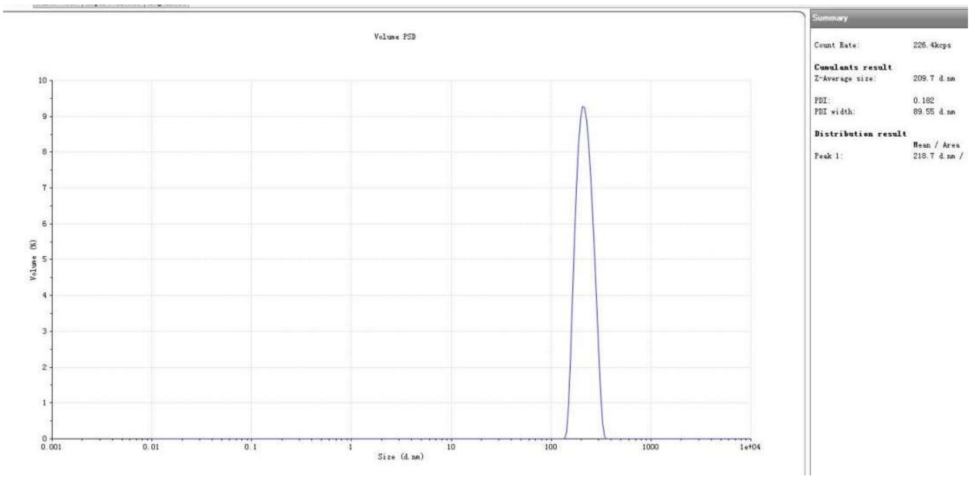

1. zein NPs (5mg/mL)

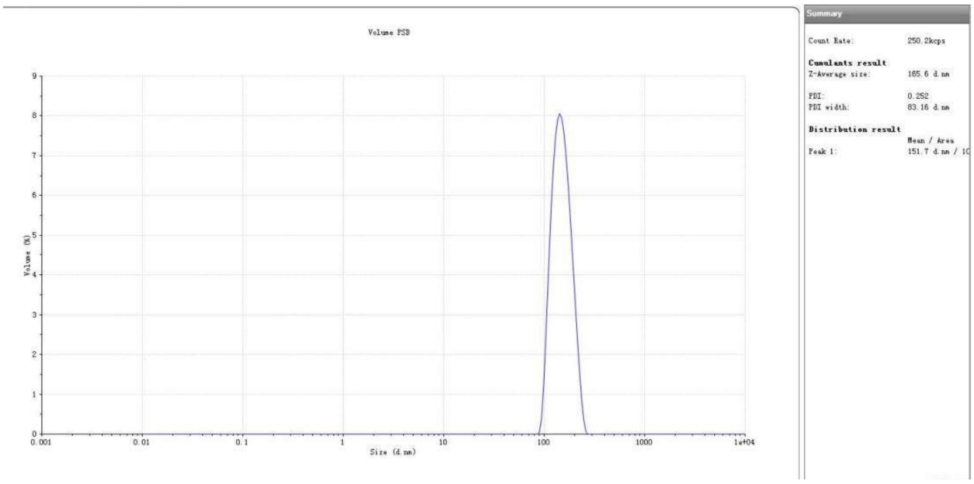

2. zein NPs (10mg/mL)

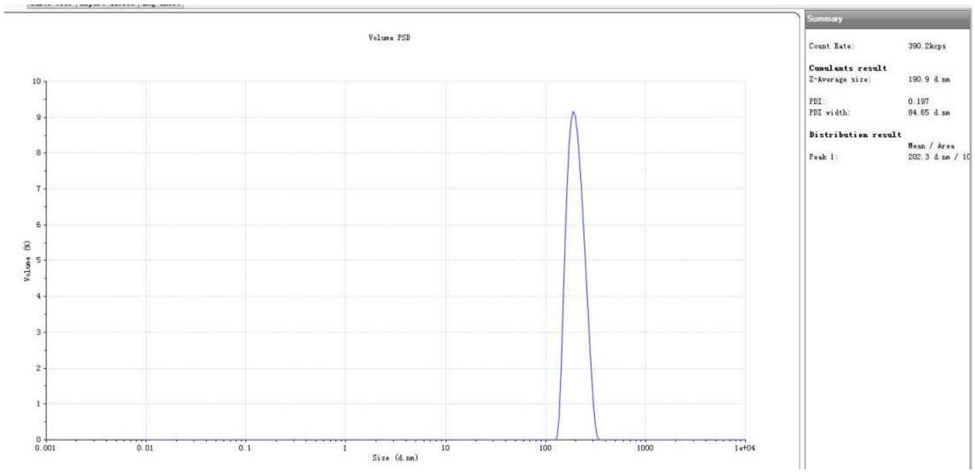

3. zein NPs (15mg/mL)

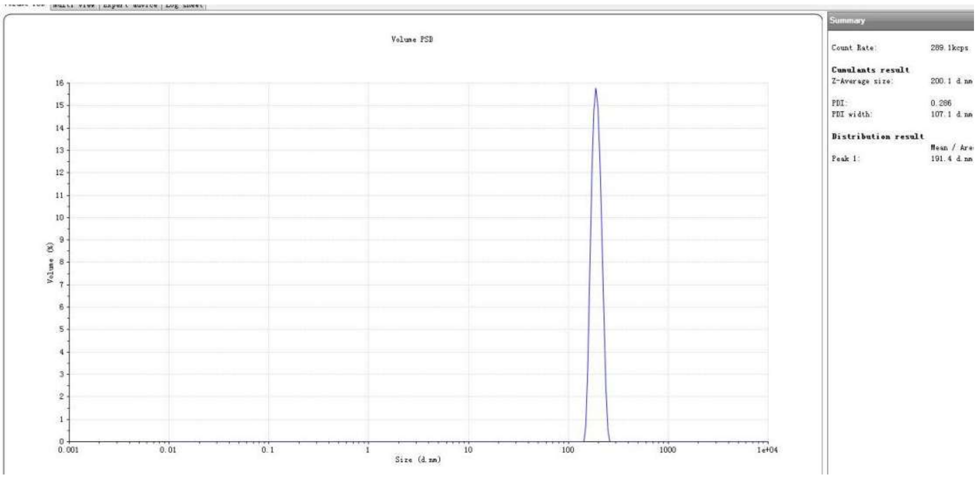

4. zein NPs (20mg/mL)

Supplementary Figure S2

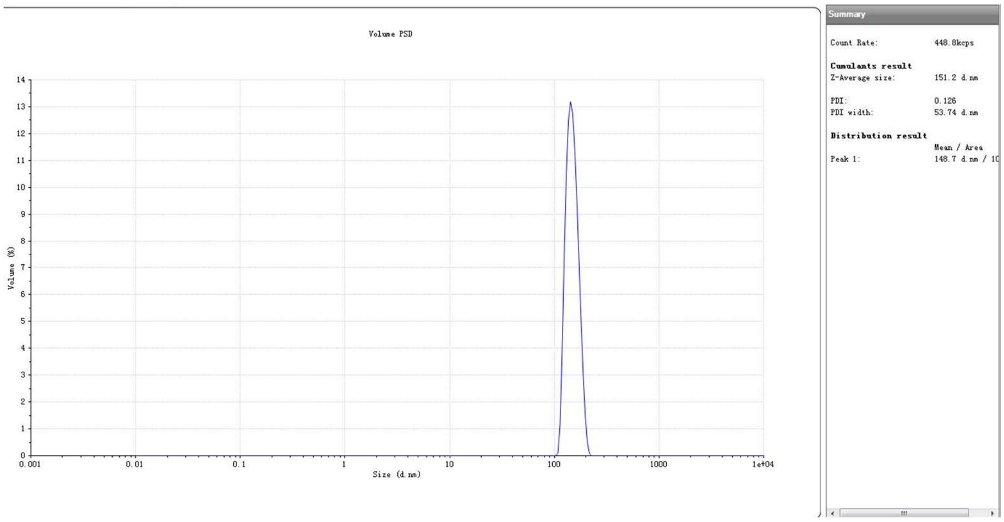

1. zein NPs (zein 10 mg/ml)

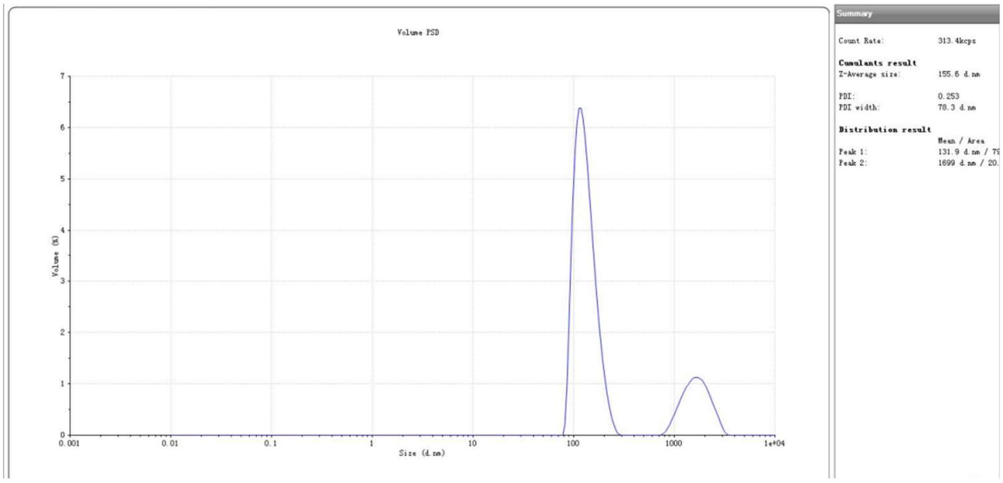

2. Z/S NPs (4:1)

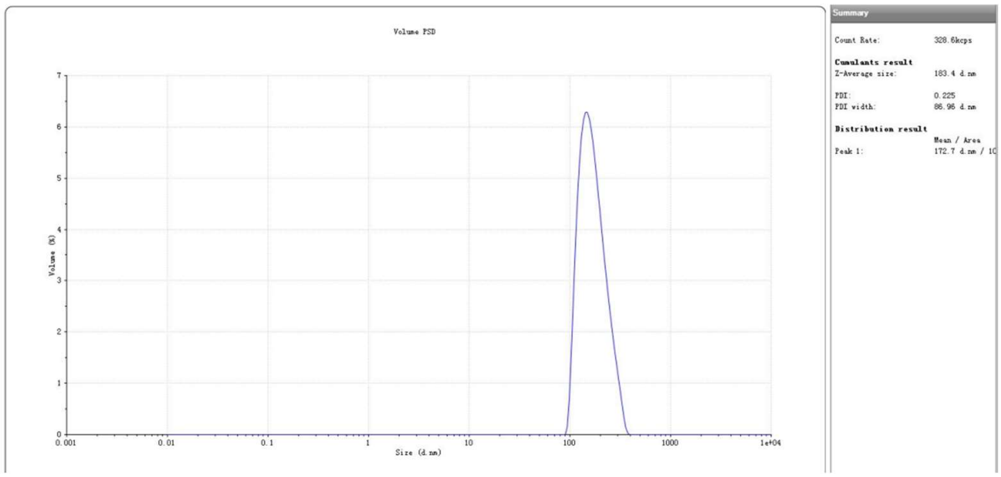

3. Z/S NPs (2:1)

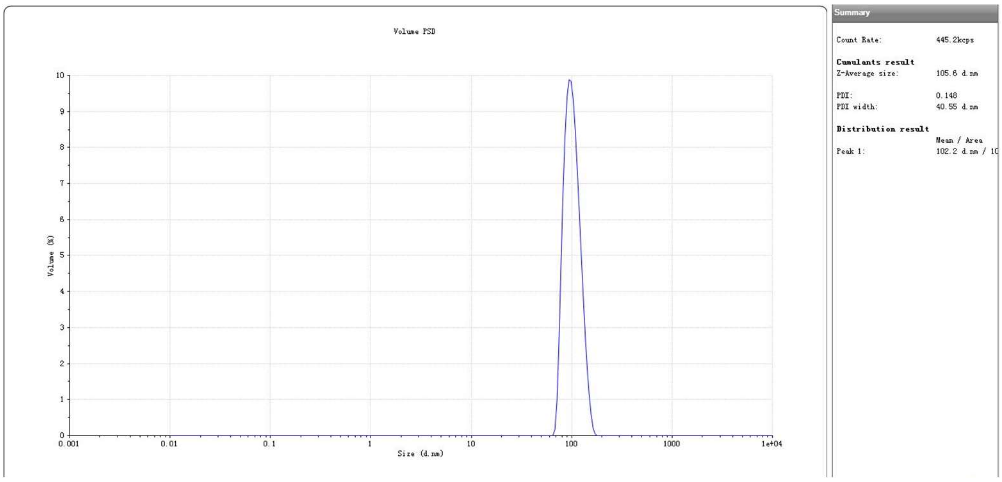

4. Z/S NPs (1:1)

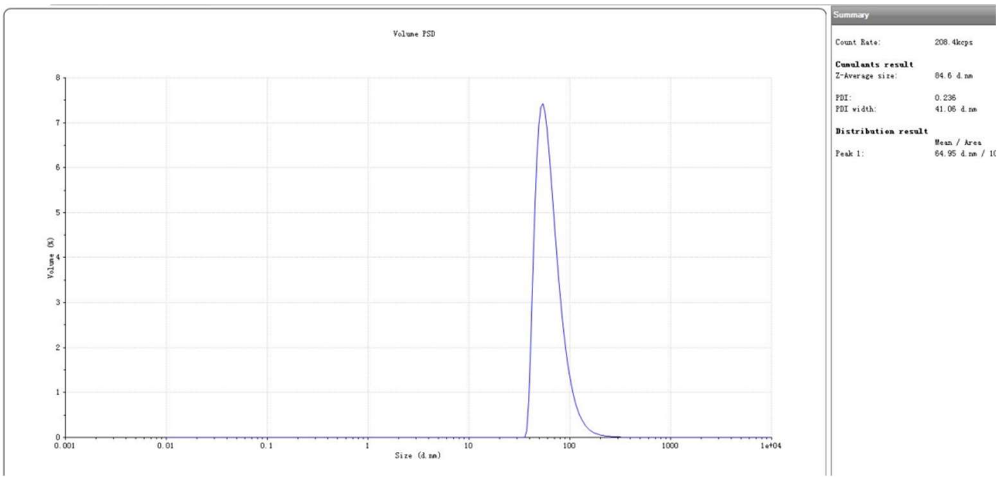

5. Z/S NPs (2:3)

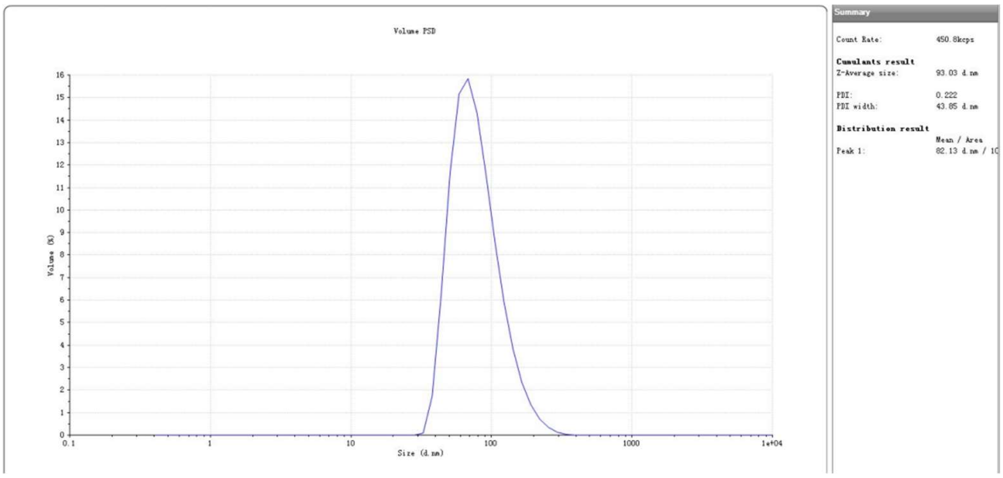

6. Z/S NPs (1:2)

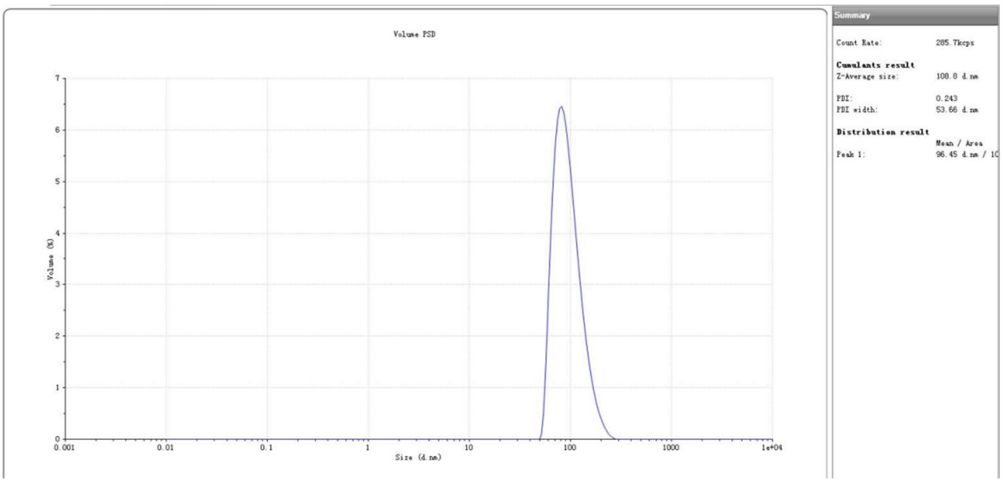

7. Z/S NPs (2:5)

Supplementary Figure S3

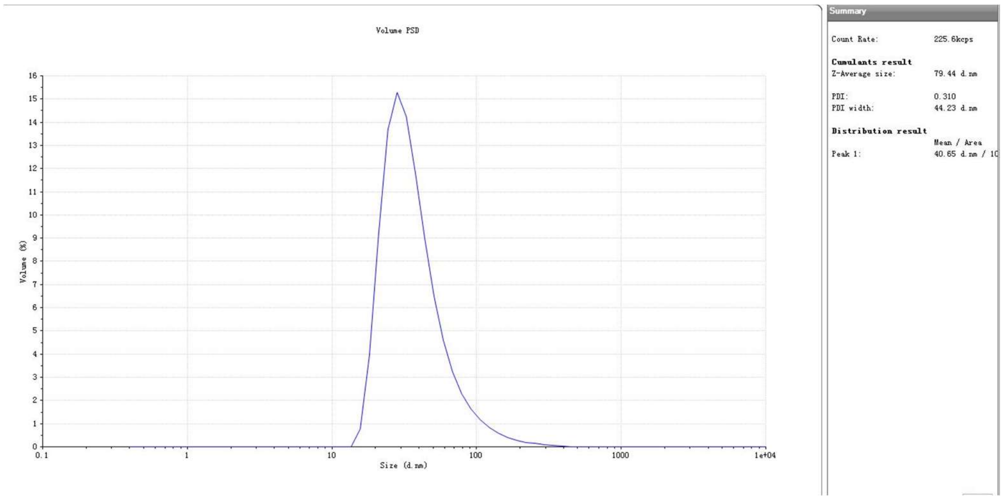

1. S/C (4:1)

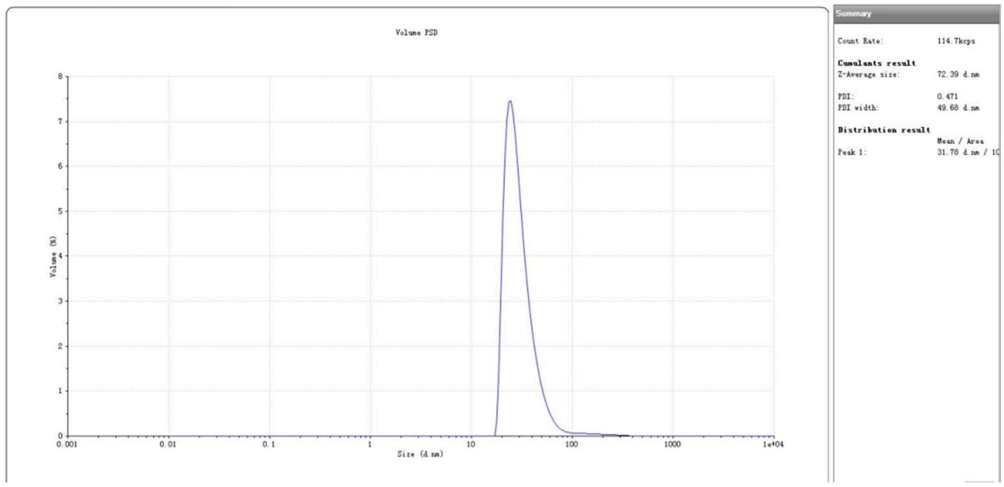

2. S/C (2:1)

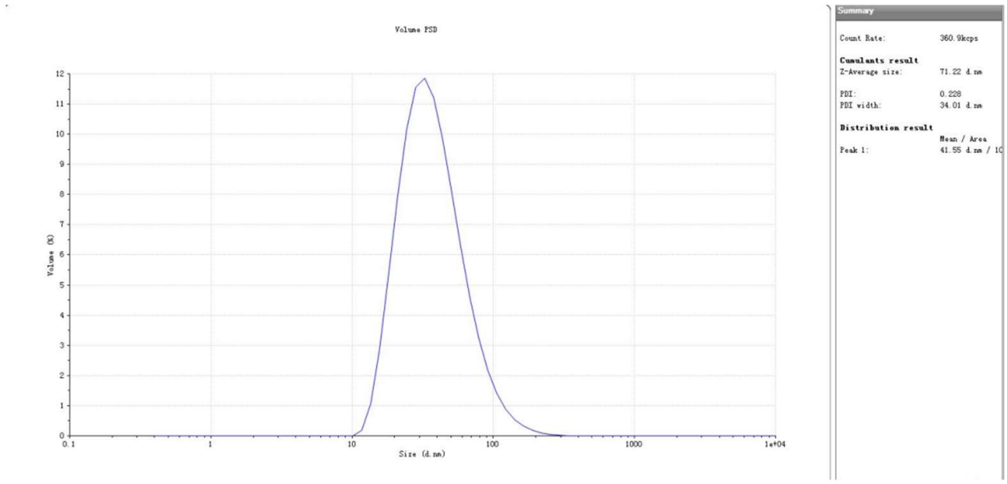

3. S/C (1:1)

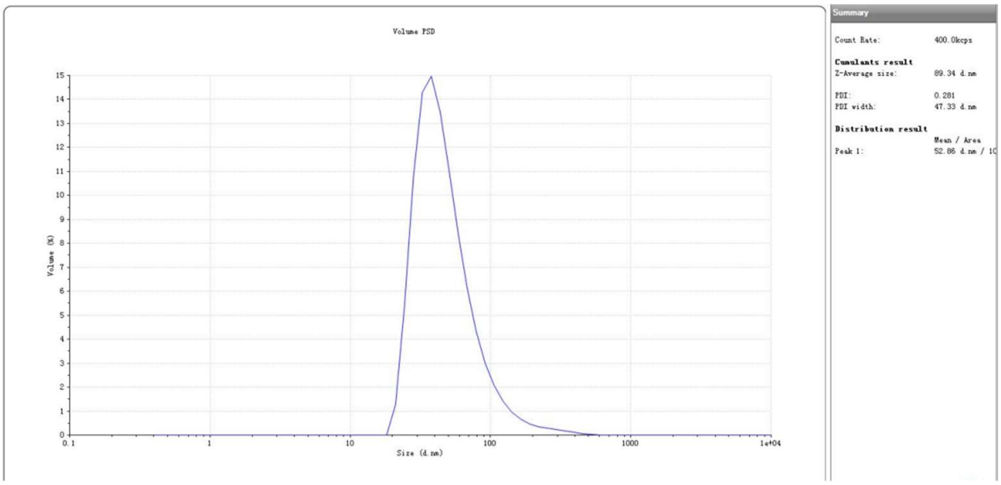

4. S/C (2:3)

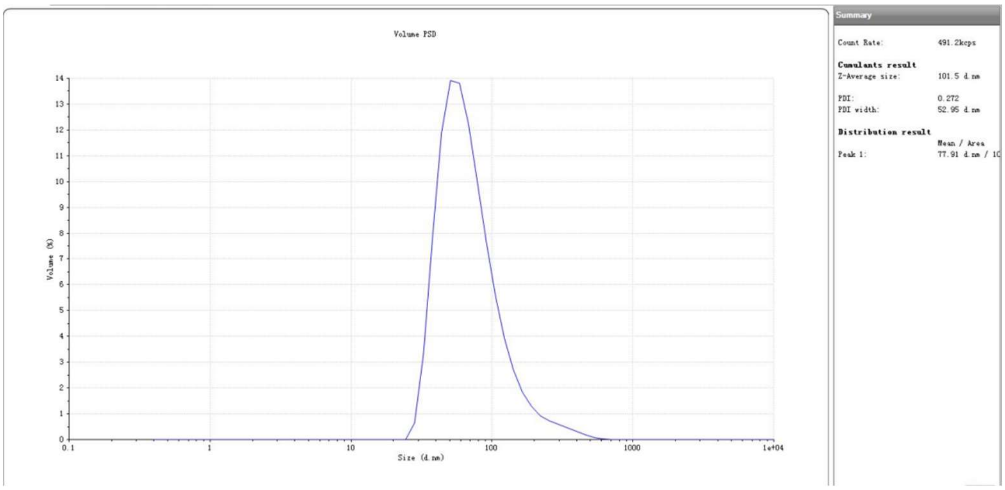

5. S/C (1:2)

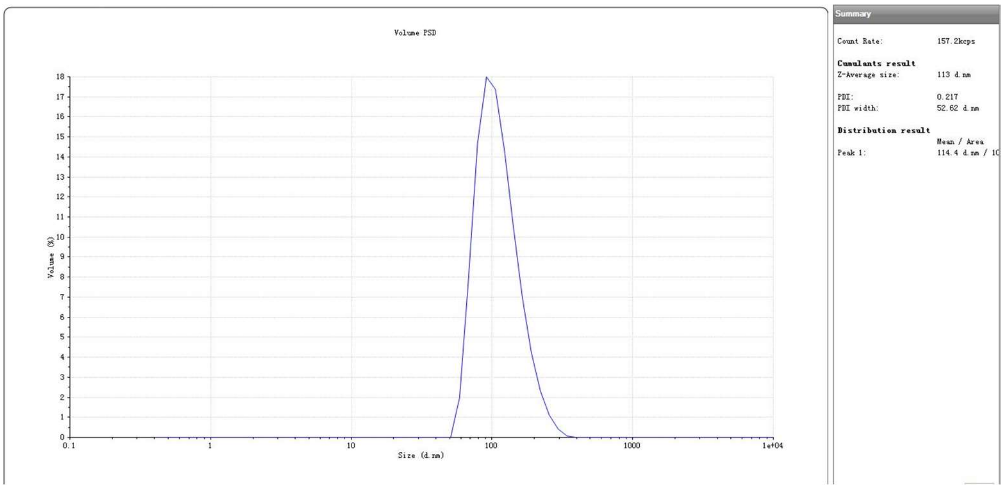

6. S/C (2:5)

Supplementary Figure S4

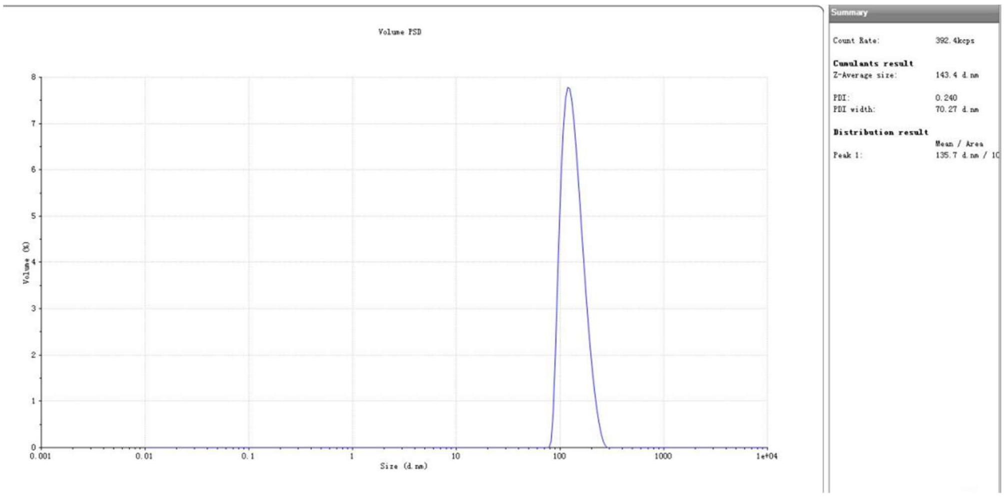

1. Z-cur NPs

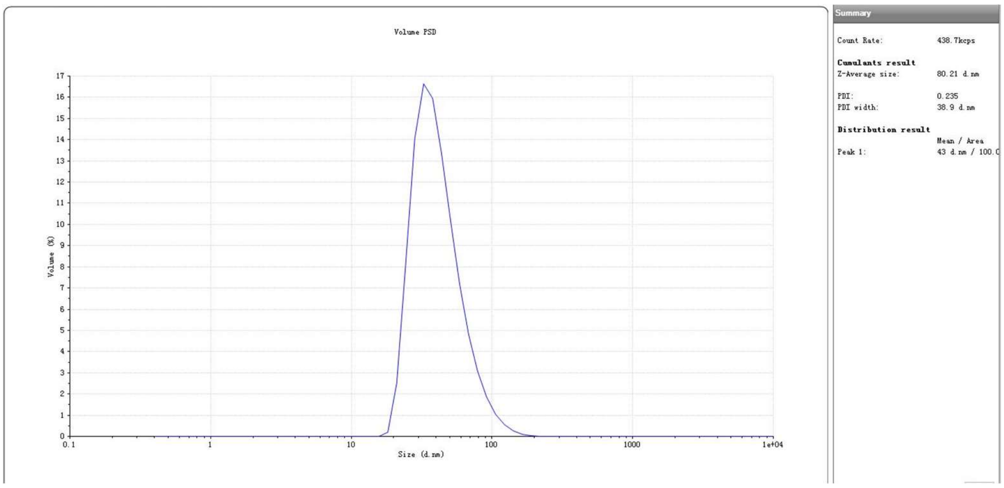

2. Z/S-cur NPs

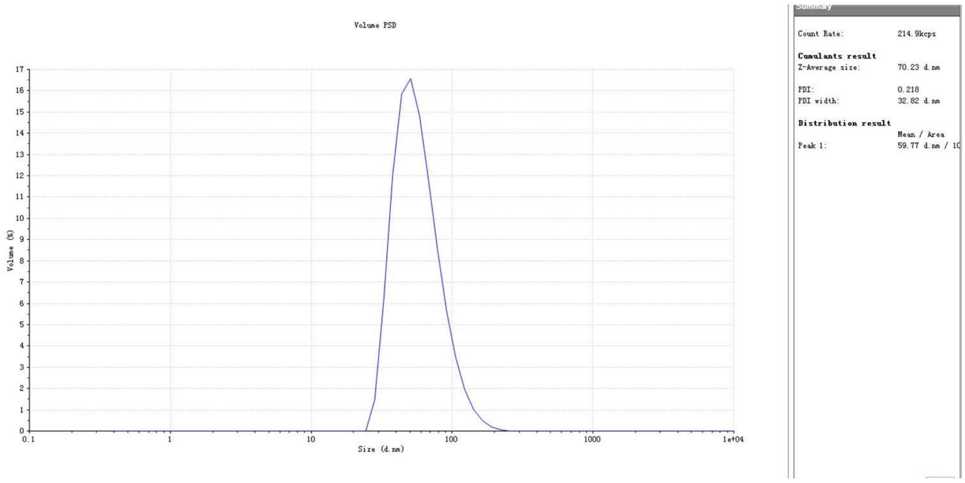

3. Z/S/C-cur NPs

Supplementary Figure S5

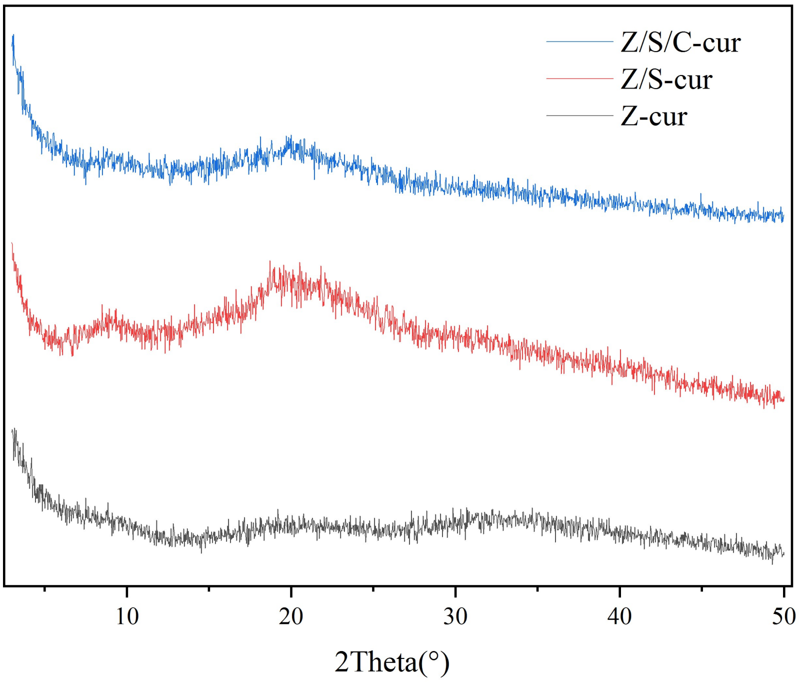

Supplement: Supplementary file 1 [file foods-12-02692-s001.zip › foods-2438731-supplementary.pdf]
